# Effect of Antioxidant Treatment on Fibrogenesis in Rats with Carbon Tetrachloride-Induced Cirrhosis

**DOI:** 10.5402/2012/762920

**Published:** 2012-04-02

**Authors:** Silvia Bona, Lidiane Isabel Filippin, Fábio Cangeri Di Naso, Cintia de David, Bruna Valiatti, Maximiliano Isoppo Schaun, Ricardo Machado Xavier, Norma Possa Marroni

**Affiliations:** ^1^Post-Graduation Medical Sciences Program, Medical School, Federal University of Rio Grande do Sul (UFRGS), 90035-903 Porto Alegre, RS, Brazil; ^2^Laboratory of Molecular Biology of Autoimmune and Infectious Disease, Hospital de Clínicas de Porto Alegre (HCPA), 90035-903 Porto Alegre, RS, Brazil; ^3^Department of Physiology, Universidade Federal do Rio Grande do Sul (UFRGS), 90040-060 Porto Alegre, RS, Brazil; ^4^Physiotherapy Course, Universidade Católica de Pelotas (UCPEL), 96010-000 Pelotas, RS, Brazil; ^5^Post-Graduation Physiology, Federal University of Rio Grande do Sul (UFRGS), 90050-170 Porto Alegre, RS, Brazil; ^6^Academic Course of Medicine, Universidade Federal de Ciências da Saúde de Porto Alegre (UFCSPA), Brazil; ^7^Post-Graduation Medical Sciences Program, Instituto de Cardiologia do Rio Grande do Sul, 91045-140 Porto Alegre, RS, Brazil; ^8^Laboratory of Molecular Biology of Autoimmune and Infectious Diseases, HCPA, 90035-903 Porto Alegre, RS, Brazil; ^9^Laboratory of Experimental Gastroenterology and Hepatology—Federal University of Rio Grande do Sul (UFRGS), 92425-900 Canoas, RS, Brazil; ^10^Laboratory of Oxidative Stress and Antioxidants—Lutheran University of Brazil (ULBRA), 92425-900 Canoas, RS, Brazil

## Abstract

*Aim*. This study aimed to assess the antioxidant activity of quercetin (Q) in an experimental model of cirrhosis induced by CCl_4_ inhalation. *Materials and Methods*. We used 25 male Wistar rats (250 g) that were divided into 3 groups: control (CO), CCl_4_, and CCl_4_ + Q. The rats were subjected to CCl_4_ inhalation (2x/week) for 16 weeks, and they received phenobarbital in their drinking water at a dose of 0.3 g/dL as a P450 enzyme inducer. Q (50 mg/Kg) was initiated intraperitoneally at 10 weeks of inhalation and lasted until the end of the experiment. Statistical analysis was by ANOVA Student Newman-Keuls (mean ± SEM), and differences were considered statistically significant when *P* < 0.05. *Results*. After treatment with quercetin, we observed an improvement in liver complications, decreased fibrosis, as analyzed by picrosirius for the quantification of collagen, and decreased levels of matrix metalloproteinase 2 (MMP-2) compared with the CCl_4_ group. It also reduced oxidative stress, as confirmed by the decrease of substances reacting to thiobarbituric acid (TBARS), the increased activity of antioxidant enzymes, and the reduced glutathione ratio and glutathione disulfide (GSH/GSSG). *Conclusion*. We suggest that the use of quercetin might be promising as an antioxidant therapy in liver fibrosis.

## 1. Introduction

Cirrhosis is an advanced stage of liver fibrosis characterized by septae and nodule formation and altered blood flow. It occurs because of the synthesis and excessive deposition of extracellular matrix (ECM) in the space of Disse along with insufficient ECM degradation, leading to a distortion of the architecture and a progressive reduction of hepatic function [1].

Wound healing is the normal response of tissue to an injury, and liver fibrosis occurs as a result of repeated cycles of injury and repair. Moreover, chronic persistent inflammation typically precedes fibrosis. Chronic liver injuries activate and transform quiescent hepatic stellate cells (HSCs) into activated myofibroblasts, which is the central pathogenic mechanism of fibrotic disorders [2].

The development of cirrhosis is usually associated with oxidative stress and lipid peroxidation (LPO) [3]. In this study, we used the carbon tetrachloride (CCl_4_) inhalation model of cirrhosis in the rat because it has several similarities with human cirrhosis [4]. CCl_4_, a widely used solvent in chemical industries, is one of the main pathways for the exposure and absorption of volatile chemicals that may be environmental contaminants, and it is well known for its hepatic and renal toxic actions. The metabolism of CCl_4_ into trichloromethyl (CCl_3_•) and peroxy trichloromethyl (•OOCCl_3_) free radicals has been reported to cause hepatotoxic effects, like fibrosis, steatosis, necrosis, and hepatocarcinoma [3].

Much effort has been devoted to developing new treatments for this disease. The only treatment currently available for severe end-stage liver disease is orthotopic liver transplantation [5].

Some compounds that have been studied as possible protectors against liver cirrhosis are known for their anti-inflammatory and antioxidant properties. Plants contain numerous polyphenols, which have been shown to reduce inflammation and thereby to increase resistance to disease [6]. Quercetin (Q), a polyphenolic flavonoid compound present in large amounts in vegetables, fruits, and tea, exhibits its therapeutic potential against many diseases, including hepatoprotection and the inhibition of liver fibrosis [7–9]. It contains a number of phenolic hydroxyl groups, which have strong antioxidant activity [10, 11]. The average intake varies between countries but is approximately 23 mg/day [10].

By increasing the endogenous antioxidant defenses, flavonoids can modulate the redox state of organisms. The major endogenous antioxidant systems include superoxide dismutase (SOD), catalase (CAT), glutathione reductase (GR), and glutathione peroxidase (GPx), which is essential for the detoxification of lipid peroxides [8, 12].

Therefore, using the carbon tetrachloride-induced liver injury model, we investigated the protective actions of the flavonoid quercetin on the progression of fibrosis and on parameters of oxidative stress.

## 2. Materials and Methods

### 2.1. Animal Experiments and Drug Treatment

Male Wistar rats weighing 250–300 g were used. The animals were caged at 22°C with 12-hour light-dark cycles and free access to food and water until the experiments were performed. All experiments were performed according to the Guiding Principles for Research Involving Animals (NAS) and the Committee of Research and Ethics in Health of the Research and Postgraduate Group of the Hospital de Clínicas de Porto Alegre.

 Experimental animals were randomly divided into a control group (*n* = 5), a cirrhotic group treated with CCl_4_ for 16 weeks (*n* = 10), and a CCl_4_ + quercetin group treated with CCl_4_ for 16 weeks and with quercetin from the 10th to 16th week (*n* = 10). A control group treated with quercetin was not necessary because previous studies by our group had demonstrated that it does not produce a significant difference compared to control animals [13].

For P450 enzymatic induction, phenobarbital (0.3 g/L) was added to the animal's drinking water seven days before the first inhalation and throughout the experiment. The CCl_4_ group was exposed to inhaled CCl_4_ twice a week (on Mondays and Fridays) inside an inhalation chamber that measured 65 × 26 × 21 cm. CCl_4_ was placed in a glass container (humidifier) attached to an air compressor and released into the chamber at a flow rate of 1 L/min. In the first three sessions, the length of gas exposure was 30 s, and the animals remained inside the chamber for another 30 s, while the compressor was turned off (waiting time). In the fourth session, the length of gas administration was increased to 1 minute, followed by another minute in the waiting mode. Subsequently, the length of gas administration and the waiting period in the chamber were increased by 30 s every three sessions, up to a peak of 5 min at 16 weeks, according to the method adapted from Cremonese et al. [14].

Quercetin (Sigma) was administered i.p. at a dose of 50 mg/kg/day [9, 13]. It was initiated at the 10th week, when histological analyses and liver function tests indicated that the animals were already cirrhotic, and was carried out until the date of sacrifice [4].

After 24 hours from the last CCl_4_ inhalation, the animals were anesthetized with 1% xylasine and 10% ketamine, and then we collected blood samples from the retro-orbital plexus. Later, the livers were removed, washed with saline, and divided into sections. A portion was preserved in 4% formalin for histological examination. The rest was frozen at −80°C for later analysis.

### 2.2. Serum Biochemical Analysis

Serum activities of aspartate aminotransferase (AST), alanine aminotransferase (ALT), alkaline phosphatase (AP), and total bilirubin (BT) were measured with routine laboratory methods of the Hospital de Clínicas de Porto Alegre.

### 2.3. Histological Analysis

For histological examination, a piece of the liver from all animals was trimmed and fixed by immersion in 10% buffered formalin for 24 hours. The blocks were dehydrated in a graded series of ethanol and embedded in paraffin wax. Serial 3 mm sections were stained with hematoxylin and eosin or picrosirius. Five sections from each sample were analyzed by two independent pathologists who had no prior knowledge of the animal groups.

### 2.4. Collagen Quantification

Collagen was determined by estimating the hydroxyproline content, an amino acid characteristic of collagen. Liver sections of 100 mg were hydrolyzed in 6 mol/L HCl for 16 h at 110°C and evaporated to dryness to remove the acid. The residue, dissolved in distilled water, was mixed with 50% isopropanol and chloramine-T solution and left for 10 min at room temperature. Finally, p-dimethylaminobenzaldehyde in 60% perchloric acid was added and heated to 60°C for 25 min [15]. The absorbance was measured at 560 nm. Hydroxyproline levels were calculated based on standard curves of 4-hydroxy-1-proline and expressed as *μ*g/mg protein.

### 2.5. Oxidative Damage Determination

Frozen tissue from each rat was homogenized in ice-cold phosphate buffer (140 mM KCl, 20 mM phosphate, pH 7.4) and centrifuged at 3,000 rpm for 10 minutes. Oxidative stress was determined by measuring the concentration of aldehydic products (MDA) by thiobarbituric acid reactive substances (TBARSs) [16]. Spectrophotometric absorbance of the supernatant at 535 nm was determined.

### 2.6. Antioxidant Enzyme Activity

#### 2.6.1. Superoxide Dismutase (SOD)

Cytosolic superoxide dismutase (SOD) (EC 1.5.1.1) was assayed at 30°C according to Misra and Fridovich [17]. The auto-oxidation rate of epinephrine, which is progressively inhibited by increasing amounts of SOD in the homogenate, was monitored spectrophotometrically at 560 nm. The amount of enzyme that inhibited 50% of epinephrine auto-oxidation was defined as 1 U of SOD activity.

#### 2.6.2. Catalase (CAT)

Catalase activity was determined by measuring the decrease in absorption at 240 nm in a reaction medium containing 50 mM phosphate buffer saline (pH 7.2) and 0.3 M hydrogen peroxide [18]. The enzyme activity was assayed spectrophotometrically at 240 nm.

#### 2.6.3. Glutathione Peroxidase (GPx)

The glutathione peroxidase (GPx) activity was determined by the oxidation rate of NADPH in the presence of reduced glutathione and glutathione reductase [19]. Sodium azide was added to inhibit catalase activity. The GPx activity was measured with a spectrophotometer at 340 nm.

### 2.7. Measurement of Intracellular Reduced Glutathione/Oxidized Glutathione (GSH/GSSG)

GSH and GSSG measurements were made according to the adapted method from Kolberg et al. [20]. Liver sections were rinsed twice with PBS and disrupted in 200 mL of 5% (w/v) metaphosphoric acid on ice. After centrifugation (16,000x g, 2 min at room temperature), cell lysates were spectrophotometrically (415 nm) assayed on a microplate reader by modification of the 5,50-dithiobis(2-nitrobenzoic acid) (DTNB)/GSSG reductase recycling method using the N-ethylmaleimide conjugating technique for GSSG sample preparation. Samples (10 mL) for both GSH and GSSG determinations were assayed in a 105 *μ*L final volume in 96-well polystyrene plates at 37°C in the presence of 10 mM DTNB, 0.17 mM *β*-NADPH (dissolved in 0.5% (w/v) NaHCO_3_ as a stabilizing agent), and 0.5 U/mL GSSG reductase.

### 2.8. Western Blot

 Protein extraction and western blotting were performed as described elsewhere [21]. The membranes were incubated with anti-MMP-2 polyclonal antibody (sc-8853, *Santa Cruz Biotechnology*). Binding to the primary antibody was detected through rabbit anti-immunoglobulin bound to HRP (DAKO A/S, Glostrup, Denmark). Protein detection was performed by chemiluminescence using a commercial kit ECL (Amersham Pharmacia Biotech, Little Chalfont, Great Britain) exposing the membrane to this commercial mixture for one minute. A cassette tape was subsequently introduced with developing film (Amersham Hyperfilm ECL, UK) for about 2 minutes.

 After washing the film, the bands were quantified by densitometry using program Scion Image 4.02 for Windows (Scion Corporation, Frederick, USA), with results being expressed in relation to control percentage (100%).

### 2.9. Statistical Analysis

The results were expressed as mean ± SEM. The data were compared by analysis of variance (ANOVA); when the analysis indicated the presence of a significant difference, the means were compared with the Student Newman-Keuls test. Significance was accepted at *P* < 0.05.

## 3. Results

### 3.1. Serum Biochemical Analysis

After 16 weeks of CCl_4_ exposure, the animals showed important alterations in enzyme markers of hepatic injury (Table 1). The CCl_4_-treated group showed a significant increase in serum total bilirubin and hepatic marker enzymes. However, the intraperitoneal administration of quercetin at 50 mg/kg attenuated this elevation of AST, ALT, ALP, and BT.

### 3.2. Histological Analysis

**Figure 1:**
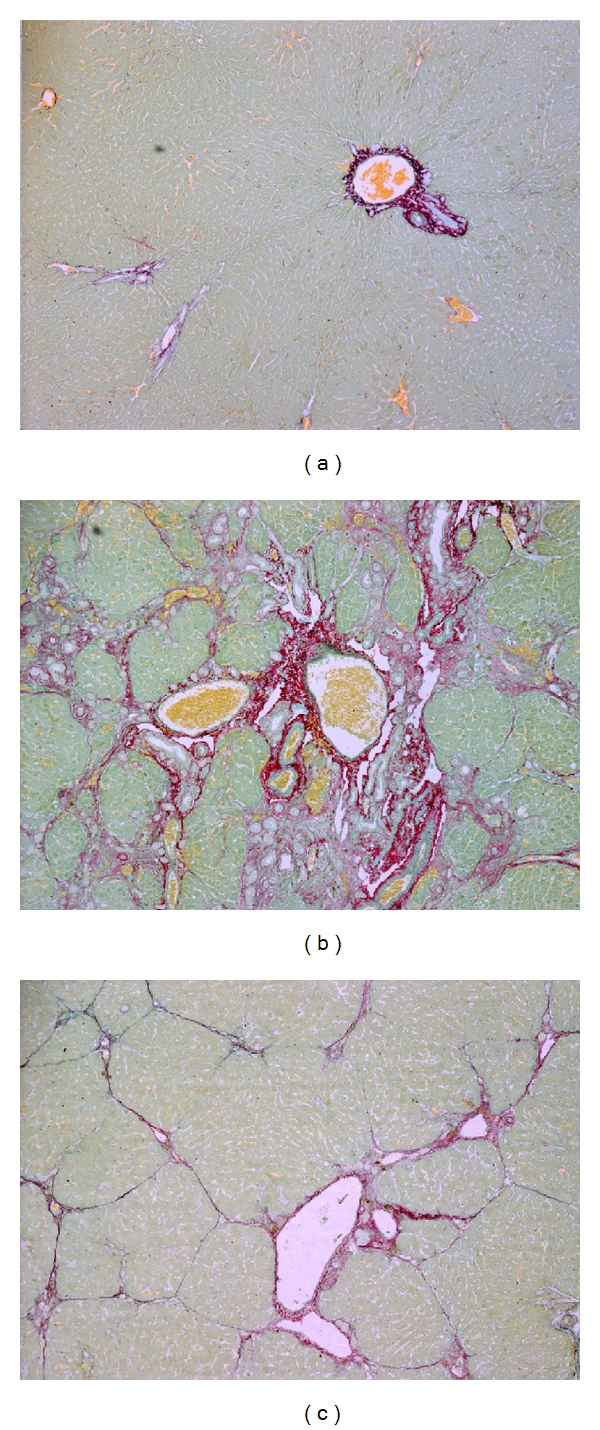
Histological analysis of liver sections by picrosirius staining. (a) Control rat liver section (100x); (b) cirrhotic rat (CCl_4_) liver section (100x); (c) cirrhotic rat treated with Q (CCl_4_ + Q) liver section (100x).

The histological analysis of liver tissue from animals in the control group (CO) showed a normal architecture of the parenchyma (Figure 1(a)). Animals with CCl_4_ exposure showed a loss of the normal architecture with the presence of regenerative nodules, cellular necrosis, and fibrosis (Figure 1(b)). In contrast, necrosis and fibrosis were minimal in animals from groups treated with quercetin (Figure 1(c)).

### 3.3. Collagen

**Figure 2:**
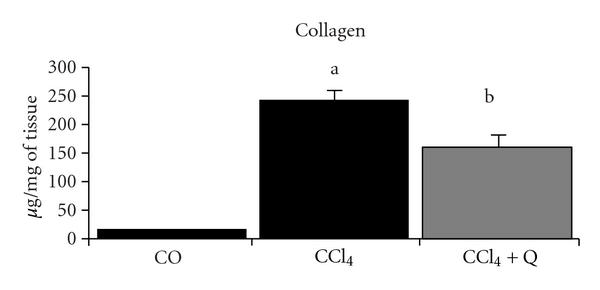
Medium values of collagen in the liver from different groups. ^a^Significant difference between the CCl_4_ group and the CO and CCl_4_ + Q groups (*P* < 0.01). ^b^Significant difference between the CCl_4_ + Q group and the CO group (*P* < 0.05).

Fibrosis, which is the final result of prolonged liver injury, was quantified by hydroxyproline analysis and expressed as liver collagen content (Figure 2). The collagen content was significantly higher in the CCl_4_-treated group. This effect was partially but significantly reduced by quercetin.

### 3.4. Lipid Peroxidation

Oxidative stress resulting from the metabolism of CCl_4_ in the liver plays a critical role in damaging the liver and promoting hepatic fibrogenesis. The MDA level was significantly higher in liver homogenates of CCl_4_-intoxicated rats compared with the control group (Table 2). Treatment with quercetin significantly reduced these levels. 

### 3.5. Antioxidant Enzymes

CCl_4_ toxicity resulted in a marked reduction of SOD, CAT, and GPx activities in liver tissues compared with the control group (Table 2). Treatment with quercetin significantly reestablished the levels of these antioxidant enzymes.

The hepatic GSH concentration decreased significantly after CCl_4_ treatment, and this decrease was attenuated by quercetin. Concomitantly, the GSSG concentration increased in the livers of CCl_4_-treated animals. The ratio of GSH to GSSH decreased when compared with the control group after CCl_4_ injection, and quercetin significantly restored this decrease at a dose of 50 mg/kg for 16 weeks (Table 2).

### 3.6. Matrix Metalloproteinase 2 (MMP-2) Expression

**Figure 3:**
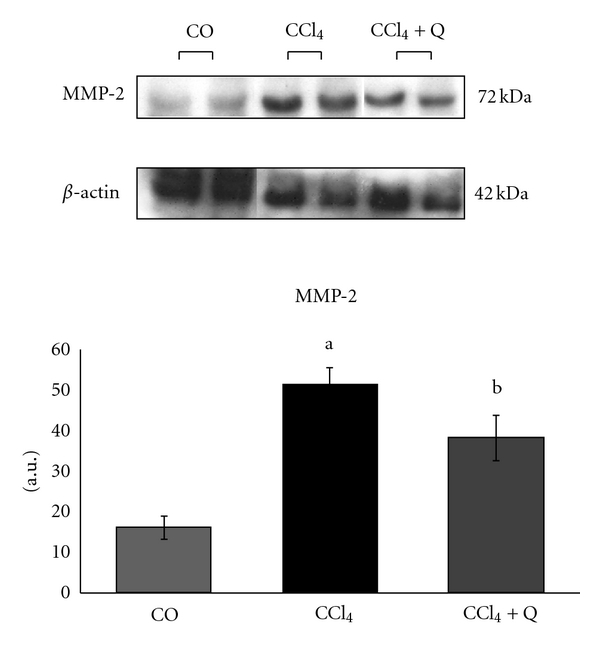
Medium values of matrix metalloproteinase 2 (MMP2) expression from the different studied groups. ^a^Significant difference between the CCl_4_ group and the CO and CCl_4_ + Q groups (*P* < 0.05). ^b^Significant difference between the CCl_4_ + Q group and the CO group (*P* < 0.05).

 Matrix Metalloproteinase 2 (MMP-2) increased significantly after CCl_4_ treatment, and a decreased of protein expression was observed after treatment with quercetin of 50 mg/kg for 16 weeks (Figure 3).

## 4. Discussion

CCl_4_ is widely used to induce hepatic fibrosis and cirrhosis in animal models. Furthermore, the liver signs observed in rats due to chronic stimulation with CCl_4_ are similar to those found in cirrhotic patients [4]. Oxidative stress plays an important role in the development of hepatic fibrosis acting on different cell types and in different signaling pathways. Consequently, antioxidants, particularly those of plant origin, have emerged as potent antifibrotic agents [8, 9]. In this study, hepatic fibrosis was successfully induced by CCl_4_ inhalation for 16 weeks. Through this hepatic fibrosis model, the effects of quercetin on hepatic fibrosis induced by CCl_4_ in rats were examined, and the treatment was initiated 8 weeks after the initiation of CCl_4_ inhalation. The flavonoid was administered at a dose previously described to have beneficial effects in rats with biliary obstruction [9, 13]. Quercetin spreads in the cell membrane and acts as a scavenger or obstructs the chain reaction of free radicals formed, thereby decreasing lipid peroxidation [22]. Similar effects were also obtained after the use of the flavonoid quercetin in rats in models of cirrhosis induced by intraperitoneal carbon tetrachloride [7] and by biliary obstruction [13].

AST and ALT are enzymes that are sensitive to hepatocellular injury. The release of large quantities of these enzymes in the bloodstream is associated with centrilobular necrosis, degeneration, and reduced performance status of the liver [23]. In this study, we observed a significant increase in the serum levels of AST, ALT, ALP, and BT after CCl_4_ inhalation. The decrease of the serum enzymes AST, ALT, ALP, and BT after treatment with quercetin demonstrated its antioxidant potential and its hepatoprotective effect.

The histological findings revealed severe liver cell damage in rats after the inhalation of CCl_4_, supporting the observed changes in biochemical assays. The presence of necrotic foci, fibrotic nodules, infiltration of lymphocytes, steatosis, and changes in liver cells are characteristics after intoxication with CCl_4_ [24]. Treatment with quercetin decreased necrosis and fibrosis. This can be considered an expression of the functional improvement of hepatocytes. These data are consistent with other studies of cirrhosis that used substances with antioxidant power, like quercetin [13], silymarin [25], and curcumin [6].

In this study, traditional methods of assessing hepatic fibrosis, such as the quantification of hydroxyproline content and histological analysis, were performed to determine the effects of quercetin on liver cirrhosis induced by CCl_4_. The determination of hydroxyproline content in liver tissue is regarded as a good method to quantify fibrosis and to evaluate the efficacy of new antifibrotic agents [26]. In this study, the exposure to CCl_4_ significantly increased the hydroxyproline content in the liver. This increase in collagen deposition was reduced after treatment with quercetin, demonstrating its effectiveness on liver fibrogenesis.

In addition to the effects of increased synthesis of collagen induction of cirrhosis also showed an increased expression of the enzyme matrix metalloproteinase 2 (MMP-2). The key enzymes in the degradation of fibrillar collagens are matrix metalloproteinase (MMP)-1 in humans and MMP-13 in rodents [27, 28]. However, during fibrogenesis, the expression of MMP-1 or MMP-13 is very limited, whereas that of MMP-2 increases [29]. Our study showed a reduction in the expression of MMP-2 after treatment with quercetin and these results are consistent with an *in vitro* study that demonstrated decreased secretion of matrix metalloproteinases (MMP-2 and MMP-9) after a 72-hour treatment with quercetin [30].

MDA is the main product of lipid peroxidation, and its concentration is generally presented as the total level of lipid peroxidation products [31]. It has been shown that MDA can activate stellate cells that produce collagen. MDA was analyzed by the TBARS assay, and its level was significantly higher in the CCl_4_-intoxicated group compared with the others. The increase in LPO could have caused the loss of structure and integrity of the cell membrane. Quercetin treatment significantly reduced the TBARS levels in liver homogenates.

Several studies have found increased LPO in rats treated with CCl_4_, whereas the activities of enzymes in the liver were decreased [7, 32] also to those found in this study. Free-radical scavenging enzymes, such as superoxide dismutase (SOD), protect the biological systems from oxidative stress. The current study showed a significant decrease in SOD activity in rats with CCl_4_-induced cirrhosis. On the other hand, there was a significant increase in SOD activity in rats treated with quercetin. Our results indicate that treatment with quercetin acts against free radicals formed during the metabolism of CCl_4_ by restoring the levels of the antioxidant enzymes SOD, CAT, and GPx and reducing LPO.

While there are several biochemical markers of oxidative stress, the ratio of reduced GSH/GSSG has been widely used as a marker of the redox state because of its sensitivity, reliability, and abundance. GSH is the main endogenous antioxidant and is responsible for the maintenance of the intracellular redox balance, detoxification of xenobiotics, and reactive oxygen species [33]. The reduction of GSH in the liver, observed in this study, may be caused by the direct requisition of GSH by GPx to scavenge the production of free radicals formed by the metabolism of CCl_4_. In this study, the administration of quercetin not only increased the levels of total hepatic GSH but also significantly improved the ratio of GSH/GSSG in the liver. Several studies indicate that quercetin has the ability to increase the GSH/GSSG and, consequently, decreases cellular oxidative damage [34].

A possible explanation for these findings is that quercetin not only acts as a direct scavenger of reactive oxygen species but may also exert actions on endogenous antioxidant defenses by modulating the intracellular signaling systems. This protection may be the result of a variety of mechanisms, including decreasing oxidative stress due to the sequestration of free radicals, promoting cell survival by modulating intracellular signals, and decreasing the toxicity of xenobiotics and carcinogens by regulating gene expression or the activity of the cytochrome P_450_ enzymes [35].

In summary, administration of quercetin seems to have a protective role in liver of rats with experimentally induced cirrhosis, as demonstrated by the reduction of fibrosis and oxidative stress.

## Figures and Tables

**Table 1:** Effect of quercetin on hepatic enzymes in CCl_4_-induced hepatic injury.

Parameters	Experimental groups		
	CO	CCl_4_	CCl_4_ + Q
AST (U/L)	119.2 ± 10.1	488.6 ± 61.7^a^	256.5 ± 34.3
ALT (U/L)	35.7 ± 3.6	235.2 ± 17.4^a^	165.9 ± 38.7^b^
AP (U/L)	66.7 ± 5.0	237.6 ± 18.5^a^	167.6 ± 17.7^b^
BT (U/L)	0.3 ± 0.0	0.8 ± 0.1^a^	0.5 ± 0.08

Results represent mean ± S.E.

^a^Significant difference between CCl_4_ group and groups CO and CCl_4_ + Q, considering *P* < 0.05.

^b^Significant difference between the CCl_4_ + Q group and group CO, considering *P* < 0.05.

**Table 2:** Effects of quercetin (Q) on lipid peroxidation and on antioxidant enzyme activities in CCl_4_-induced hepatic injury.

Parameters	Experimental groups		
	CO	CCl_4_	CCl_4_ + Q
TBARS (nmol/mg protein)	0.19 ± 0.001	0.37 ± 0.02^a^	0.28 ± 0.02^b^
SOD (U SOD/mg protein)	3.86 ± 0.28	1.41 ± 0.22^a^	3.36 ± 0.29
CAT (p mol/mg protein)	0.62 ± 0.04	0.27 ± 0.02^a^	0.55 ± 0.02
GPx (mmol/min/mg protein)	0.27 ± 0.02	0.19 ± 0.01^a^	0.23 ± 0.01
GSH (mmol/mg protein)	362.1 ± 46.63	230.66 ± 35.09^a^	371.6 ± 33.8
GSSG (nmol/mg protein)	40.63 ± 1.87	84.46 ± 3.45^a^	49.96 ± 3.74
GSH/GSSG (Ratio)	8.91 ± 1.06	2.74 ± 0.42^a^	7.43 ± 0.80

Results represent mean ± S.E.

^a^Significant difference between CCl_4_ group and groups CO, CCl_4_ + Q, considering *P* < 0.05.

^b^Significant difference between CCl_4_ + Q group and groups CO, considering *P* < 0.05.
